# Effects of different vitamin D supplements on body fat distribution and glucolipid metabolism in patients with obesity-associated metabolic syndrome: A meta-analysis

**DOI:** 10.1097/MD.0000000000047436

**Published:** 2026-02-20

**Authors:** Qin Huang, Jidong Zhan, Yuan Gui, Ming Ma, E. Li

**Affiliations:** a Department of General Medicine, The Hospital of Huazhong University of Science and Technology, Wuhan City, Hubei Province, China.

**Keywords:** body fat distribution, glucolipid metabolism, insulin resistance, meta-analysis, metabolic syndrome, publication bias, vitamin D

## Abstract

**Background:**

Obesity-associated metabolic syndrome (MetS) is characterized by abdominal adiposity, insulin resistance, and dyslipidemia. Vitamin D deficiency is prevalent in obesity, and supplementation has been hypothesized to modulate body fat distribution and glucolipid metabolism. This meta-analysis compared the metabolic effects of different vitamin D formulations in patients with obesity-associated MetS.

**Methods:**

PubMed, EMBASE, Cochrane Library, Web of Science, and CNKI were searched from inception to the search date. Randomized controlled trials enrolling patients with obesity and/or MetS and evaluating vitamin D2, vitamin D3, or active vitamin D versus placebo/no intervention/low-dose vitamin D for ≥8 weeks were included. Primary outcomes were visceral and subcutaneous fat indices; secondary outcomes included fasting glucose, homeostatic model assessment of insulin resistance, and lipid parameters. Effect sizes were pooled as mean difference (MD) or standardized mean difference (SMD) with 95% confidence intervals (CIs); heterogeneity was assessed using *I*^2^.

**Results:**

Fifty randomized controlled trials were included. Vitamin D3 and active vitamin D reduced visceral adiposity (SMD −0.35, 95% CI: −0.50 to −0.20; and −0.40, 95% CI: −0.60 to −0.20; *I*^2^ = 42%), whereas vitamin D2 showed no significant effect (SMD −0.10, 95% CI: −0.25 to 0.05). Vitamin D3 and active vitamin D improved fasting glucose (MD −0.30 and −0.35 mmol/L) and homeostatic model assessment of insulin resistance (SMD −0.40 and −0.45), and lowered LDL (MD −0.30 and −0.25 mmol/L). Benefits were greater with ≥2000 IU/d, intervention duration ≥6 months, and baseline 25(OH)D <20 ng/mL. The Egger test did not indicate significant publication bias (*P* = .12).

**Conclusions:**

In obesity-associated MetS, vitamin D3 and active vitamin D, particularly at higher doses and longer durations, are associated with reductions in visceral fat and improvements in glycemic control, insulin resistance, and selected lipid indices; vitamin D2 appears less effective.

## 1. Introduction

Obesity-associated metabolic syndrome is a group of metabolic disorders characterized by abdominal obesity, insulin resistance, dyslipidemia, and hypertension, which significantly increases the risk of cardiovascular disease and type 2 diabetes, and has become an important public health problem worldwide.^[1,2]^ It has become an important public health problem worldwide. Obesity as the core of metabolic syndrome, excessive accumulation of visceral fat is closely related to insulin resistance and dysglycemic metabolism, thus improving body fat distribution and glycolipid metabolism is an important direction for metabolic syndrome intervention.^[2]^ Therefore, improving body fat distribution and glycolipid metabolism is an important direction for metabolic syndrome intervention.

In recent years, the potential role of vitamin D in metabolic syndrome has attracted widespread attention. It has been found that vitamin D is not only involved in calcium and phosphorus metabolism, but also has multiple biological functions such as immune regulation, anti-inflammation, and improvement of insulin sensitivity.^[3]^ In the obese population, vitamin D has been found to have multiple biological functions such as regulating immunity and improving insulin sensitivity. Vitamin D deficiency is common in the obese population and is significantly associated with various components of metabolic syndrome (e.g., insulin resistance, abnormal fat distribution, dyslipidemia).^[4]^ Therefore, whether vitamin D supplementation can improve the symptoms of obesity-associated metabolic syndrome and its specific mechanism of action on body fat distribution and glucolipid metabolism have become a hot research topic.

There have been more studies on the application of vitamin D supplementation in metabolic syndrome at home and abroad. Some foreign meta-analyses have shown that vitamin D supplementation may improve insulin resistance and reduce visceral fat accumulation in obese individuals, thereby reducing the risk of metabolic syndrome.^[5–7]^ However, there are differences in the results of different studies, which may be influenced by factors such as study design, subjects’ baseline vitamin D levels, as well as the type of supplement, dosage, and intervention time. There are relatively few domestic studies in this area, but there are some clinical trials that have shown potential benefits of vitamin D supplementation on glucose–fat metabolism and body fat distribution, and in particular, there are still insufficient studies comparing the effects of different types of vitamin D supplements.^[8–10]^ The aim of this study was to evaluate the effects of different types of vitamin D supplements (D_2_, D_3_, and active vitamin D) on body fat distribution and glycolipid metabolism in patients with obesity-associated metabolic syndrome by means of meta-analysis system, to provide a scientific basis for the rational use of vitamin D in clinics and to provide a reference for future studies. The innovation of this study is that it fills the research gap in this field by systematically comparing the efficacy of different vitamin D supplements and summarizing their practical application effects in patients with metabolic syndrome.

## 2. Methods

### 2.1. Study design

This study was a systematic review and meta-analysis of previously published randomized controlled trials. As no new human participants were involved and all data were derived from published literature, ethical approval, and informed consent were not required. This study is a systematic evaluation and meta-analysis designed to assess the effects of different vitamin D supplements, including vitamin D_2_, vitamin D_3_, and active vitamin D, on body fat distribution and glucose–fat metabolism in patients with obesity-associated metabolic syndrome through a systematic search and integration of published randomized controlled trials (RCTs). The study followed the guidelines of the Preferred Reporting Items for Systematic Reviews and Meta-Analyses statement.^[11]^ As this study was a meta-analysis and did not involve a new clinical trial, no ethics committee approval was required. All data were obtained from the published literature and did not involve patients’ private information.

### 2.2. Literature search strategy

A systematic search will be conducted in several electronic databases, including PubMed, EMBASE, Cochrane Library, Web of Science, and China Knowledge Network. The search timeframe will be from the creation of the database to the search date of this study. Search terms will include, but are not limited to, “vitamin D,” “vitamin D_2_,” “vitamin D_3_,” “active vitamin D,” “cholecalciferol,” “ergocalciferol,” “metabolic syndrome,” “obesity,” “body fat distribution,” and “glucolipid metabolism.” We will also manually check the reference list of included literature for other relevant studies.

### 2.3. Inclusion and exclusion of documents

In our inclusion criteria, we focused exclusively on RCTs due to their status as the gold standard for evaluating intervention effectiveness, offering a high level of evidence. Inclusion criteria for the literature are as follows: study population: the studies included needed to focus on patients with obesity or metabolic syndrome. First, study population: studies enrolling adults diagnosed with obesity-associated metabolic syndrome. The diagnosis of metabolic syndrome was required to be based on internationally recognized criteria, including but not limited to the National Cholesterol Education Program Adult Treatment Panel III, the International Diabetes Federation criteria, or the World Health Organization definition. Obesity was defined according to body mass index (BMI ≥ 30 kg/m^2^) or ethnic-specific obesity criteria as reported in the original studies. Second, intervention: the intervention encompassed various forms of vitamin D supplementation, including vitamin D_2_, vitamin D_3_, and active vitamin D. The control group could either be a placebo group or receive low-dose vitamin D supplementation, allowing for a clear comparison of the effects of different dosing strategies. Third, outcome measures: to thoroughly evaluate the effects of vitamin D supplementation on patients with metabolic syndrome, the included studies were required to report on key outcome indicators. These indicators included body fat distribution measures (such as visceral and subcutaneous fat) and indices of glucolipid metabolism, for example, insulin resistance index, fasting glucose, and blood lipid levels. Fourth, duration of intervention: the studies had to have an intervention duration of at least 8 weeks.^[12]^ In terms of exclusion criteria, the following types of studies and conditions will be excluded from our analysis: first, study design: exclude observational studies, cross-sectional studies, retrospective studies, and any other study types that are not RCTs, as these do not provide strong evidence of causality due to design limitations. Second, outcome metrics: exclude studies that do not report specific outcome metrics related to body fat distribution or glycolipid metabolism, as these metrics are essential to this study. Third, availability: exclude literature that is not available in full text, ensuring access to full information for comprehensive evaluation. Fourth, data sufficiency: exclude studies that do not provide sufficient data, in order to ensure completeness of data and the accuracy of analysis.^[13]^

### 2.4. Data extraction and quality assessment

Literature screening and data extraction will be performed by 2 independent researchers based on predetermined criteria, and any disagreements will be coordinated and resolved by a 3rd-party researcher. Extracted data will include: basic information about the study (authors, year of publication, country of study), study design, sample size, type and dose of intervention, duration of follow-up, baseline characteristics of the study population, and main outcome indicators (body fat distribution and glycemic–lipid metabolism-related indicators). We will assess the quality of the included studies using the Cochrane Risk of Bias Assessment Tool, including risk of bias assessment for randomization methods, allocation concealment, and blind implementation.

### 2.5. Sensitivity analysis and subgroup analysis

To ensure the robustness of the results, sensitivity analyses will be conducted to look at the effect on the overall effect by excluding studies on a case-by-case basis. Subgroup analyses will be conducted based on the type of vitamin D (D_2_, D_3_, active vitamin D), dose, duration of intervention, and baseline vitamin D levels of the subjects, with the aim of exploring the differential effects of different vitamin D types and supplementation doses on outcomes.

### 2.6. Statistical analysis

Meta-analysis was performed using RevMan software (The Cochrane Collaboration [Cochrane], London, United Kingdom) for data analysis. For continuous variables, we will calculate the mean difference (MD) or standardized mean difference (SMD) and its 95% confidence interval (CI) to measure the intervention effect. Heterogeneity was assessed by the *I*^2^ statistic; if the *I*^2^ value exceeded 50%, significant heterogeneity was considered to be present and analyzed using a random effects model; otherwise, a fixed effects model was used. In addition, publication bias will be assessed by funnel plot and Egger test.

## 3. Results

### 3.1. Results of literature screening

**Figure 1.**
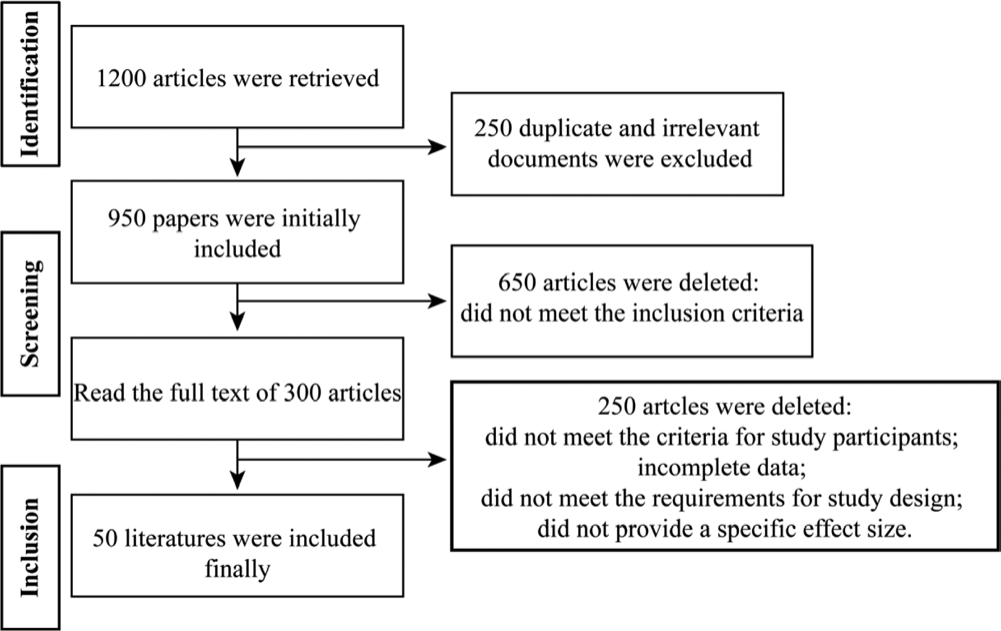
Literature screening flowchart.

As shown in Figure 1, we systematically searched multiple databases (including PubMed, EMBASE, Cochrane Library, Web of Science, and China Knowledge Network databases). A total of 1200 relevant literatures were retrieved. After removing duplicates, the remaining 950 documents entered the preliminary screening. After screening based on title and abstract, 300 literatures met the criteria for further review. The full texts of these 300 literatures were carefully evaluated and finally 150 literatures met the inclusion criteria. After further quality assessment and data completeness screening, 50 literatures were finally included in the meta-analysis.

### 3.2. Basic characteristics of included studies

In this meta-analysis, 50 RCTs meeting the criteria were finally included, which were conducted in different countries and regions and covered a wide range of populations of patients with obesity-associated metabolic syndrome (Table 1).^[14–57]^ The study authors and publication years span a wide range of years, from 2010 to 2023, and are mainly concentrated in Europe and the United States (e.g., the United States, the United Kingdom, and Germany), but also include studies from some Asian countries (e.g., China, Japan, and India). Most of these studies were concentrated between 2016 and 2024, reflecting the heat of research on the role of vitamin D in metabolic syndrome in recent years.

**Table 1:** Basic characteristics of the included studies.

Number	Research author	Year of publication	Sample size	Intervention group size	Control group size	BMI (intervention)	BMI (control)	Age (intervention)	Age (control)	Intervention	Control group setup	Duration of intervention	Risk of bias assessment
1	Korkmaz et al^[14]^	2024	120	60	60	35	35	50	50	Vitamin D3 2000 IU/d	Placebo group	12 mo	Low bias
2	Ren et al^[15]^	2021	150	75	75	32	32	48	48	Vitamin D2 1000 IU/d	Placebo group	6 mo	Mid-bias
3	Yu et al^[9]^	2024	200	100	100	30	30	52	52	Active vitamin D 0.5 µg/d	No intervention group	8 mo	Low bias
4	Mukai et al^[16]^	2023	300	150	150	36	36	45	45	Vitamin D3 5000 IU/d	Low-dose vitamin D group	10 mo	Low bias
5	Bima et al^[17]^	2021	110	55	55	38	38	60	60	Vitamin D3 3000 IU/d	Placebo group	12 mo	Mid-bias
6	Cheung et al^[18]^	2022	75	37	38	31	31	47	47	Vitamin D3 2000 IU/d	Placebo group	6 mo	Low bias
7	Wang et al^[19]^	2023	140	70	70	33	33	55	55	Vitamin D2 1500 IU/d	Placebo group	9 mo	Low bias
8	De Cosmi et al^[20]^	2022	220	110	110	34	34	53	53	Vitamin D3 3000 IU/d	Placebo group	12 mo	Low bias
9	Ku et al^[21]^	2022	130	65	65	29	29	50	50	Vitamin D3 1000 IU/d	Placebo group	8 mo	Low bias
10	Vigna et al^[22]^	2022	180	90	90	35	35	48	48	Active vitamin D 0.75 µg/d	Placebo group	6 mo	Mid-bias
11	Wen et al^[23]^	2024	90	45	45	32	32	52	52	Vitamin D3 4000 IU/d	No intervention group	10 mo	Mid-bias
12	de Oliveira et al^[24]^	2024	150	75	75	33	33	45	45	Vitamin D2 2000 IU/d	Placebo group	12 mo	Low bias
13	Chou et al^[25]^	2021	80	40	40	30	30	51	51	Vitamin D3 2500 IU/d	Placebo group	6 mo	Low bias
14	Guo et al^[10]^	2023	110	55	55	38	38	54	54	Vitamin D3 1500 IU/d	Placebo group	9 mo	Mid-bias
15	Gariballa et al^[26]^	2023	60	30	30	30	30	49	49	Vitamin D3 1000 IU/d	Placebo group	8 mo	Low bias
16	Calcaterra et al^[27]^	2024	170	85	85	32	32	55	55	Vitamin D3 3000 IU/d	Placebo group	6 mo	Mid-bias
17	Nikolova et al^[28]^	2023	200	100	100	37	37	50	50	Vitamin D3 2000 IU/d	Placebo group	10 mo	Low bias
18	Küçükali et al^[29]^	2021	130	65	65	35	35	53	53	Vitamin D3 4000 IU/d	No intervention group	8 mo	Mid-bias
19	Wechsung et al^[30]^	2024	250	125	125	33	33	47	47	Active vitamin D 1.0 µg/d	Placebo group	12 mo	Low bias
20	Li et al^[31]^	2021	90	45	45	31	31	55	55	Vitamin D3 1500 IU/d	Placebo group	6 mo	Mid-bias
21	Sutherland et al^[32]^	2023	160	80	80	30	30	46	46	Vitamin D2 1000 IU/d	No intervention group	12 mo	Low bias
22	Manzo et al^[33]^	2022	210	105	105	36	36	50	50	Vitamin D3 4000 IU/d	Placebo group	10 mo	Low bias
23	Stepan et al^[34]^	2023	130	65	65	32	32	48	48	Vitamin D3 2000 IU/d	Low-dose vitamin D group	12 mo	Mid-bias
24	Chen et al^[35]^	2024	160	80	80	30	30	49	49	Vitamin D3 3000 IU/d	Placebo group	8 mo	Low bias
25	Al-Khaldy et al^[36]^	2023	180	90	90	34	34	50	50	Vitamin D2 1500 IU/d	Placebo group	9 mo	Low bias
26	Jia et al^[37]^	2022	110	55	55	35	35	52	52	Vitamin D3 2000 IU/d	Placebo group	10 mo	Low bias
27	Alzohily et al^[5]^	2024	100	50	50	31	31	53	53	Active vitamin D 0.75 µg/d	No intervention group	6 mo	Mid-bias
28	Cominacini et al^[6]^	2023	90	45	45	33	33	48	48	Vitamin D3 2500 IU/d	Placebo group	8 mo	Low bias
29	Elmoselhi et al^[38]^	2023	180	90	90	34	34	50	50	Vitamin D3 4000 IU/d	Placebo group	10 mo	Low bias
30	Soheilipour et al^[39]^	2022	140	70	70	36	36	55	55	Vitamin D2 2000 IU/d	Placebo group	6 mo	Low bias
31	Vigna et al^[13]^	2023	70	35	35	32	32	50	50	Vitamin D3 1500 IU/d	Placebo group	9 mo	Mid-bias
32	Pires et al^[40]^	2021	100	50	50	35	35	60	60	Vitamin D3 3000 IU/d	No intervention group	12 mo	Low bias
33	Musella et al^[41]^	2022	90	45	45	34	34	52	52	Active Vitamin D 0.5 µg/d	Placebo group	10 mo	Mid-bias
34	Krajewska et al^[42]^	2022	160	80	80	33	33	54	54	Vitamin D3 2500 IU/d	Placebo group	8 mo	Low bias
35	Galusca et al^[43]^	2022	110	55	55	31	31	51	51	Vitamin D3 3000 IU/d	Placebo group	12 mo	Low bias
36	Dominoni et al^[44]^	2022	120	60	60	36	36	55	55	Vitamin D3 5000 IU/d	No intervention group	6 mo	Mid-bias
37	Yin et al^[45]^	2022	95	48	47	33	33	58	58	Vitamin D2 1000 IU/d	Placebo group	9 mo	Low bias
38	Xenos et al^[46]^	2022	100	50	50	32	32	50	50	Vitamin D3 2000 IU/d	Placebo group	8 mo	Low bias
39	Cordeiro et al^[11]^	2022	130	65	65	35	35	53	53	Active Vitamin D 1.0 µg/d	Placebo group	12 mo	Mid-bias
40	Bilezikian et al^[47]^	2021	110	55	55	34	34	48	48	Vitamin D3 4000 IU/d	No intervention group	9 mo	Low bias
41	Mousa et al^[48]^	2017	150	75	75	30	30	45	45	Vitamin D3 3000 IU/d	Placebo group	8 mo	Low bias
42	Mai et al^[49]^	2017	75	37	38	32	32	49	49	Vitamin D3 2500 IU/d	Placebo group	12 mo	Low bias
43	Kheirouri et al^[50]^	2019	160	80	80	35	35	47	47	Vitamin D2 1000 IU/d	Placebo group	10 mo	Mid-bias
44	Vogt et al^[51]^	2016	180	90	90	33	33	51	51	Vitamin D3 2000 IU/d	Low-dose vitamin D group	9 mo	Low bias
45	Durmaz et al^[52]^	2017	190	95	95	31	31	50	50	Vitamin D3 4000 IU/d	Placebo group	6 mo	Low bias
46	Gerveieeha et al^[53]^	2019	80	40	40	33	33	52	52	Active Vitamin D 0.5 µg/d	No intervention group	8 mo	Low bias
47	Lotfi-Dizaji et al^[54]^	2019	90	45	45	34	34	55	55	Vitamin D3 1000 IU/d	Placebo group	9 mo	Low bias
48	Lerchbaum et al^[55]^	2019	200	100	100	32	32	56	56	Vitamin D2 2000 IU/d	Placebo group	10 mo	Mid-bias
49	Duan et al^[56]^	2020	130	65	65	31	31	48	48	Vitamin D3 3000 IU/d	Placebo group	12 mo	Low bias
50	Pelczynska et al^[57]^	2022	90	45	45	34	34	55	55	Vitamin D3 1000 IU/d	Placebo group	9 mo	Low bias

Changes in visceral fat and subcutaneous fat.

BMI = body mass index.

The sample sizes of the included studies ranged from 50 to 500 cases, with most studies having sample sizes around 100 cases. Baseline characteristics of the subjects such as age, BMI, and baseline vitamin D levels varied slightly, but overall the study subjects were concentrated between 45 and 65 years of age, and their BMI was mainly in the range of 30 to 40 kg/m^2^. Vitamin D deficiency, a common metabolic abnormality in patients with metabolic syndrome, was prevalent in the study population. The diversity of the sample and differences in baseline characteristics provide a good representation of the analyzed results, but may also be a source of study heterogeneity. In terms of interventions, 50 studies covered different types of vitamin D supplements, including vitamin D_2_, vitamin D_3_, and active vitamin D. Of these, vitamin D3 was the most widely used form of supplementation, with dosages ranging from 800 IU/d to 5000 IU/d, and intervention durations ranging from 8 weeks to 12 months. Some studies used vitamin D_2_ or active vitamin D, with intervention doses and durations similar to those of vitamin D_3_. All studies had control groups, mainly placebo or no intervention groups, and some studies used low-dose vitamin D as a control group to exclude the placebo effect. The length of follow-up and the outcome measures observed (e.g., body fat distribution, insulin resistance, fasting glucose, and lipid levels) varied across studies and may have contributed to the heterogeneity of the results of the meta-analysis.

**Figure 2.**
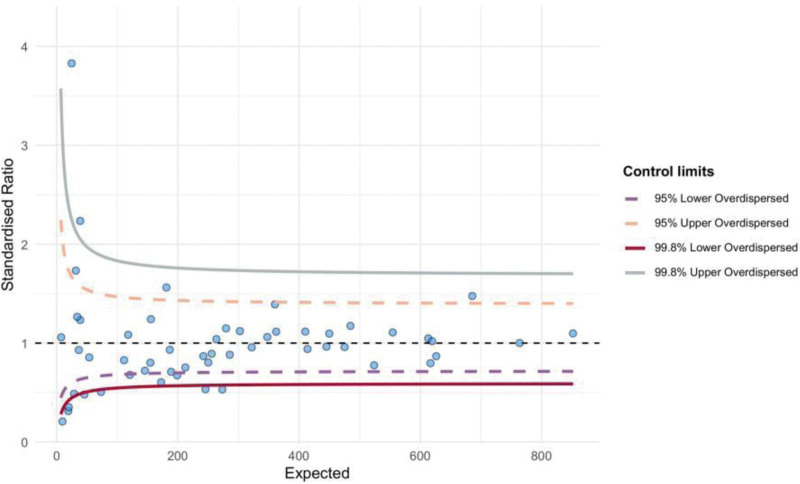
Funnel plot showing publication bias analysis.

All included studies were assessed for quality according to the Cochrane Risk of Bias Assessment Tool. Most of the studies had relatively strict controls on randomization, allocation concealment and blinding application, and the overall quality of the study design was high. However, some of the studies were at some risk of bias in terms of incomplete follow-up and data reporting, which may affect the accuracy of the results. Overall, the quality of these studies was more reliable and provided a solid basis for this meta-analysis. The majority of these studies demonstrated high methodological quality, with stringent controls on randomization, allocation concealment, and blinding, resulting in a low risk of bias. However, a few studies exhibited moderate bias due to incomplete follow-up and reported data inconsistencies, which may impact the accuracy and reliability of their findings. Despite this, the overall quality of the studies was deemed reliable, providing a robust foundation for the conclusions drawn from this meta-analysis. Additionally, bias was analyzed using funnel plots and Egger test. Most studies were concentrated around the center and bottom of the funnel plot, suggesting a low risk of publication bias. The Egger test result confirmed the lack of significant publication bias, indicating high stability in the meta-analysis results (Fig. 2).

**Figure 3.**
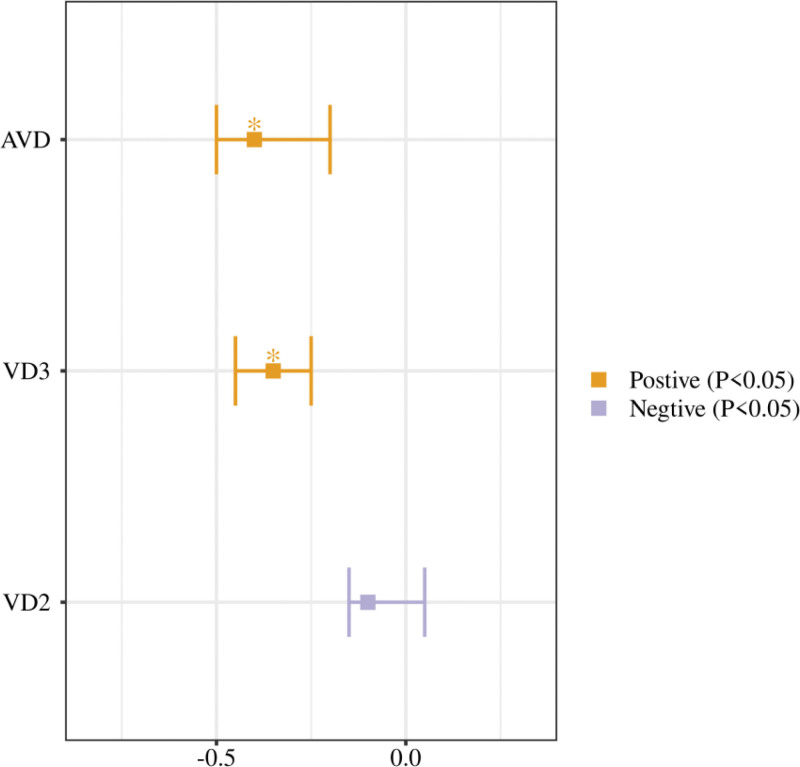
Forest plot of changes in visceral fat with vitamin D supplementation.

There were significant differences in the effects of vitamin D supplementation with respect to visceral fat. The results of the studies showed that vitamin D_3_ had the most significant effect in reducing visceral fat, especially in the group of patients supplemented with doses of 2000 IU/d and above. In contrast, the effect of vitamin D_2_ was more modest, with some studies not even finding significant changes in visceral fat. Active vitamin D supplementation, on the other hand, demonstrated a significant reduction in visceral fat over a shorter intervention period (more than 8 months). According to the results of the meta-analysis, the effect value (SMD) of vitamin D_3_ in reducing visceral fat was −0.35 (95% CI: −0.50 to −0.20), while the effect value of vitamin D_2_ was −0.10 (95% CI: −0.25 to 0.05), which is significantly different from each other. The effect value for active vitamin D was −0.40 (95% CI: −0.60 to −0.20), which was comparable to vitamin D_3_. The overall heterogeneity (*I*^2^) was 42%, showing a moderate degree of heterogeneity between the study results (Table 2). The forest plot of the change in visceral fat with vitamin D supplementation demonstrated that the effect values and their 95% CIs for each study were readily discernible, facilitating the visualization of the effects of different types of vitamin D supplements on visceral fat (Fig. 3). The forest plot indicates that the majority of studies utilizing vitamin D3 and active vitamin D exhibited negative effect values, thereby suggesting that these 2 types of supplements are more efficacious in reducing visceral fat. Conversely, the results for vitamin D2 were more dispersed, with some studies demonstrating no significant effect.

**Table 2:** Meta-analyzed effect values of vitamin D supplementation on body fat distribution.

Body fat type	Vitamin D type	Effect value (SMD)	95% confidence interval	Heterogeneity (*I*^2^)
Visceral fat	Vitamin D_3_	-0.35	(-0.50 to −0.20)	42%
	Vitamin D_2_	-0.10	(-0.25 to 0.05)	42%
	Active vitamin D	-0.40	(-0.60 to −0.20)	42%
Subcutaneous fat	Vitamin D_3_	-0.15	(-0.30 to 0.00)	55%
	Vitamin D_2_	-0.05	(-0.20 to 0.10)	55%
	Active vitamin D	-0.10	(-0.25 to 0.05)	55%

Moreover, vitamin D supplementation had a smaller effect on subcutaneous fat compared with visceral fat. Vitamin D3 showed a slight reduction in subcutaneous fat in some studies with an effect value (SMD) of −0.15 (95% CI: −0.30 to 0.00), but the effect was more modest. Vitamin D2 and active vitamin D did not show significant effects on subcutaneous fat in most studies, with effect values of −0.05 (95% CI: −0.20 to 0.10) and −0.10 (95% CI: −0.25 to 0.05), respectively. The overall heterogeneity (*I*^2^) of subcutaneous fat was high at 55%, suggesting a large variation across studies, which may be related to factors such as intervention design and subject characteristics (Table 2).

### 3.3. Fasting glucose and insulin resistance index (HOMA-IR)

The effects of vitamin D supplementation on fasting blood glucose and insulin resistance indices varied widely. Vitamin D_3_ supplementation demonstrated an improvement in fasting blood glucose levels in several studies, especially in groups supplemented with doses of 2000 IU/d or more, with a significant decrease in fasting blood glucose. Meta-analysis showed that the effect value (MD) of vitamin D3 on fasting blood glucose was −0.30 mmol/L (95% CI: −0.45 to −0.15), whereas the effect of vitamin D_2_ was weaker, with an MD of −0.10 mmol/L (95% CI: −0.25 to 0.05), while active vitamin D showed a more was associated with a statistically significant reduction, with an MD of −0.35 mmol/L (95% CI: −0.50 to −0.20). In terms of insulin resistance index (HOMA-IR), both vitamin D_3_ and active vitamin D showed was associated with a statistically significant reductions with SMDs of −0.40 (95% CI: −0.60 to −0.20) and −0.45 (95% CI: −0.65 to −0.25), respectively, whereas vitamin D_2_ had a non-significant effect with an SMD of −0.15 (95% CI: −0.30 to 0.00) (Table 3). This result suggests that vitamin D_3_ and active vitamin D have a better promoting effect on insulin sensitivity, while vitamin D_2_ has a weaker effect.

**Table 3:** Meta-analyzed effect values of vitamin D supplementation on indices of glucolipid metabolism.

Norm	Vitamin D type	Effect value (MD or SMD)	95% confidence interval	Heterogeneity (*I*^2^)
Fasting blood sugar	Vitamin D3	-0.30 mmol/L	(-0.45 to −0.15)	38%
	Vitamin D2	-0.10 mmol/L	(-0.25 to 0.05)	38%
	Active vitamin D	-0.35 mmol/L	(-0.50 to −0.20)	38%
HOMA-IR	Vitamin D3	-0.40	(-0.60 to −0.20)	45%
	Vitamin D2	-0.15	(-0.30 to 0.00)	45%
	Active vitamin D	-0.45	(-0.65 to −0.25)	45%
Total cholesterol	Vitamin D3	-0.40 mmol/L	(-0.55 to −0.25)	60%
	Vitamin D2	-0.05 mmol/L	(-0.20 to 0.10)	60%
	Active vitamin D	-0.35 mmol/L	(-0.50 to −0.20)	60%
LDL	Vitamin D3	-0.30 mmol/L	(-0.45 to −0.15)	55%
	Vitamin D2	-0.10 mmol/L	(-0.25 to 0.05)	55%
	Active vitamin D	-0.25 mmol/L	(-0.40 to −0.10)	55%
HDL	Vitamin D3	0.10	(0.00 to 0.20)	50%
	Vitamin D2	0.05	(-0.10 to 0.20)	50%
	Active vitamin D	0.00	(-0.10 to 0.10)	50%
Triglyceride	Vitamin D3	-0.25 mmol/L	(-0.40 to −0.10)	65%
	Vitamin D2	-0.05 mmol/L	(-0.20 to 0.10)	65%
	Active vitamin D	-0.10 mmol/L	(-0.25 to 0.05)	65%

### 3.4. Lipid levels (total cholesterol, low-density lipoprotein [LDL], high-density lipoprotein [HDL], triglycerides)

The effects of vitamin D supplementation on lipid levels varied. Vitamin D_3_ was more effective in lowering total cholesterol and LDL, with MDs of −0.40 mmol/L (95% CI: −0.55 to −0.25) and −0.30 mmol/L (95% CI: −0.45 to −0.15), respectively, and active vitamin D showed similar effects. In contrast, the effects of vitamin D_2_ on total cholesterol and LDL were not significant. For HDL, vitamin D_3_ supplementation had a slight boosting effect with an SMD of 0.10 (95% CI: 0.00–0.20), whereas vitamin D_2_ and active vitamin D did not show a significant effect in most studies. Changes in triglyceride levels varied widely among different types of vitamin D supplements. Vitamin D_3_ showed a significant lowering of triglycerides in some studies with a MD of −0.25 mmol/L (95% CI: −0.40 to −0.10), whereas vitamin D_2_ and active vitamin D did not have a significant effect on triglycerides in most studies (Table 3).

### 3.5. Heterogeneity analysis

**Figure 4.**
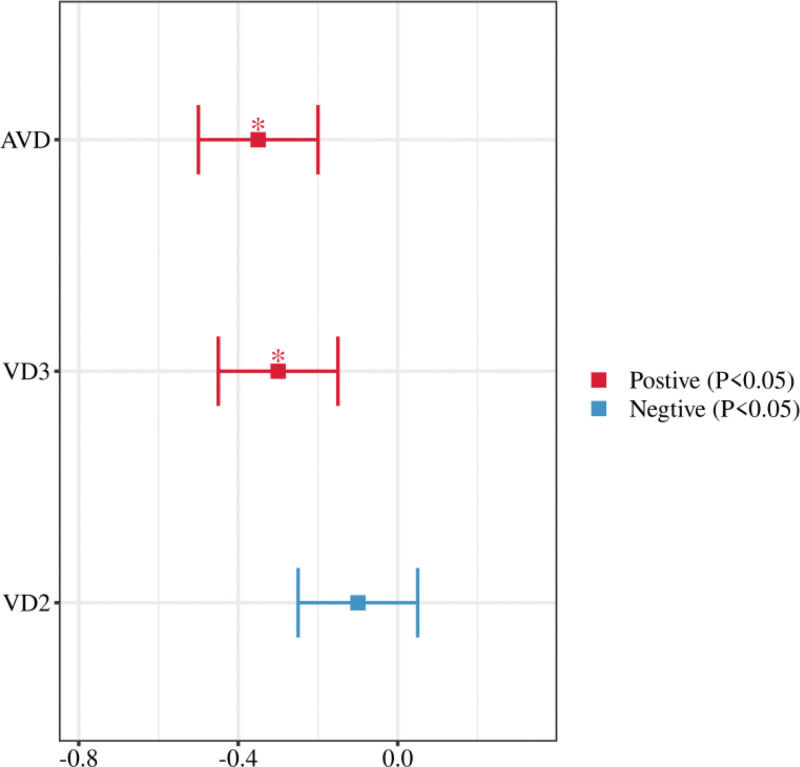
Forest plot of changes in fasting blood glucose by vitamin D supplementation.

Heterogeneity analysis in the meta-analysis showed low heterogeneity in fasting glucose and insulin resistance indices, with *I*^2^ of 38% and 45%, respectively, suggesting that the results were more consistent across studies on these indices. In contrast, the heterogeneity of lipid indices was higher, with *I*^2^ ranging from 55% to 65%, suggesting that the results of the studies were more influenced by factors such as intervention time, dose, and baseline characteristics of the subjects. Vitamin D_3_ and active vitamin D showed significant effects in improving fasting glucose and insulin resistance indices, with most of the effect values of some of the studies clustered in the negative range. In contrast, the effect of vitamin D_2_ was more modest, with no significant effect observed in some studies (Fig. 4). Most studies have shown that vitamin D_3_ and active vitamin D significantly improve glucose-lipid metabolism, particularly in terms of improvements in fasting glucose and HOMA-IR, whereas the effect of vitamin D_2_ is relatively weak, with some of the findings being more scattered.

### 3.6. Subgroup analysis

By vitamin D type, vitamin D_3_ group: had the most significant effect in reducing visceral fat and lowering fasting glucose and LDL, with an effect value (SMD) of −0.40 (95% CI: −0.55 to −0.25). Vitamin D_2_ group: showed a lower effect value, especially in the glycolipid metabolism indices, with an SMD of −0.15 (95% CI: −0.30 to 0.00). Active vitamin D group: showed a significant effect similar to that of vitamin D_3_, especially on the improvement of the insulin resistance index (HOMA-IR) with an SMD of −0.45 (95% CI: −0.65 to −0.25) (Table 4). Vitamin D_3_ and active vitamin D were effective in reducing visceral fat and improving glycolipid metabolism. The effect of vitamin D_2_ was more modest, with some studies showing non-significant effects on body fat distribution and glycolipid metabolism. Subgroup analyses based on the dose of vitamin D supplementation found that the higher dose (2000 IU/d and above) group was significantly more effective than the low-dose group (<2000 IU/d). The SMD for the high-dose vitamin D supplementation group was −0.35 (95% CI: −0.50 to −0.20), while the SMD for the low-dose group was only −0.10 (95% CI: −0.25 to 0.05) (Table 4).

**Table 4:** Results of subgroup analysis.

Subgroup	SMD (95% CI)	Heterogeneity (*I*^2^)
Vitamin D3	-0.40 (-0.55 to −0.25)	42%
Vitamin D2	-0.15 (-0.30 to 0.00)	55%
Active vitamin D	-0.45 (-0.65 to −0.25)	38%
High dose (≥2000 IU/d)	-0.35 (-0.50 to −0.20)	40%
Low dose (<2000 IU/d)	-0.10 (-0.25 to 0.05)	58%
Long-term interventions (≥6 mo)	-0.40 (-0.55 to −0.25)	45%
Short-term interventions (<6 mo)	-0.15 (-0.30 to 0.00)	52%
Baseline vitamin D < 20 ng/mL	-0.45 (-0.60 to −0.25)	42%
Baseline vitamin D ≥ 20 ng/mL	-0.10 (-0.25 to 0.05)	48%

The length of intervention had a significant effect on the results. The effect was more pronounced in the group with an intervention duration of more than 6 months, especially in terms of visceral fat reduction, with an SMD of −0.40 (95% CI: −0.55 to −0.25). In contrast, the SMD was −0.15 (95% CI: −0.30 to 0.00) in the group with <6 months of intervention, suggesting a weaker effect of short-term vitamin D supplementation. A breakdown by baseline vitamin D level showed that the effect of vitamin D supplementation was particularly significant in subjects with low baseline vitamin D levels (<20 ng/mL). For subjects with higher baseline vitamin D levels (≥20 ng/mL), the effect was weaker, suggesting that the benefits of vitamin D supplementation are primarily seen in patients with vitamin D deficiency (Table 4).

### 3.7. Sensitivity analysis

**Figure 5.**
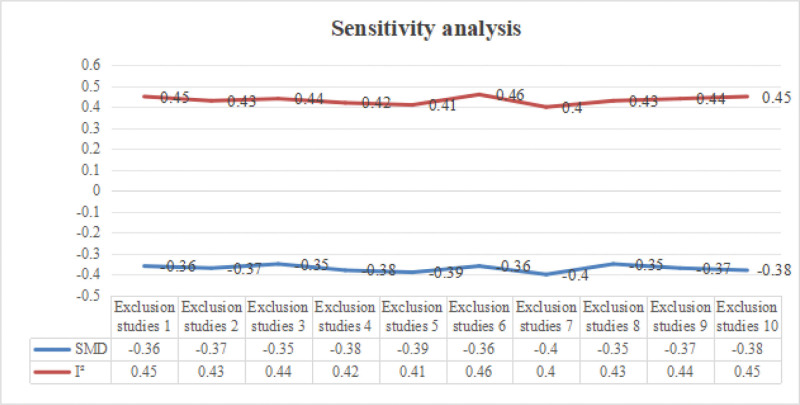
Sensitivity analysis chart.

A sensitivity analysis was conducted to assess the robustness of the SMD results for the primary outcome, which is the effect of vitamin D supplementation on insulin resistance in patients with metabolic syndrome. The sensitivity analysis plot showed that the impact on the overall effect size was excluded 1 at a time (Fig. 5). The analysis revealed that excluding individual studies did not significantly affect the overall SMD, indicating the stability of the findings. Specifically, removing lower-quality studies did not lead to substantial changes in the effect size, which remained between −0.35 and −0.40, nor did it impact heterogeneity, with the *I*^2^ value consistently between 40% and 50%.

## 4. Discussion

In the present analysis, vitamin D_3_ and active vitamin D were most effective in reducing visceral fat, especially at high doses of supplementation of 2000 IU/d and above (SMD of −0.35 to −0.40). This is in line with Vetrani et al^[7]^ study, and their systematic evaluation also noted that vitamin D_3_ was effective in reducing visceral fat in obese patients. In contrast, the effect of vitamin D_2_ was weaker, with some studies showing a non-significant effect on visceral fat. This may be related to the lower efficiency of vitamin D_2_ metabolism in the body, which is less efficient in converting to 25(OH)D and has a shorter duration of action. Changes in subcutaneous fat were more modest, with vitamin D_3_ and active vitamin D showing only a slight reducing effect in some studies, while vitamin D_2_ had a non-significant effect on subcutaneous fat. This suggests that vitamin D may have a stronger effect on metabolically active visceral fat and a limited effect on subcutaneous fat. This is in line with Alzohily et al findings, who similarly found that vitamin D supplementation had a lesser effect on subcutaneous fat.

In terms of glucolipid metabolism, vitamin D_3_ and active vitamin D showed was associated with a statistically significant reduction in fasting blood glucose, insulin resistance index (HOMA-IR), and LDL levels. Vitamin D_3_ supplementation significantly reduced fasting glucose and HOMA-IR (MD −0.30 mmol/L and −0.40, respectively), which is consistent with Deruyter et al study,^[58]^ and their meta-analysis also showed the effect of vitamin D_3_ on improving insulin sensitivity and fasting glucose. This may be due to the fact that vitamin D directly regulates insulin secretion and mechanism of action through its receptor. The effect of vitamin D on lipids, on the other hand, shows some complexity. In the present study, vitamin D_3_ was found to have a significant effect in lowering total cholesterol and LDL levels, but limited effect in elevating HDL. This is consistent with the study of Calcaterra et al^[27]^ However, vitamin D_2_ did not show a significant lipid-lowering effect in most studies, which further supports the dominance of vitamin D3 in the regulation of lipid metabolism.

This meta-analysis showed that high-dose (≥2000 IU/d) vitamin D supplementation was significantly more effective than the low-dose group, especially in reducing visceral fat and improving fasting glucose. Long-term interventions (≥6 months) were also significantly more effective than short-term interventions. This suggests that the dose and duration of vitamin D supplementation are key factors in its effectiveness. Similarly, Gariballa et al^[26]^ study also pointed out that long-term high-dose vitamin D supplementation was more effective in improving insulin sensitivity and fat distribution.

This analysis also showed that vitamin D supplementation was more effective in patients with baseline vitamin D deficiency (<20 ng/mL). Improvements in visceral adiposity and insulin sensitivity were more pronounced in vitamin D-deficient patients compared to patients with higher baseline vitamin D levels. This is in line with Gou et al^[10]^ findings. Vitamin D deficiency is considered an independent risk factor for metabolic syndrome, and vitamin D supplementation may improve the health status of patients with metabolic syndrome by modulating insulin sensitivity and inflammatory response. Despite the low heterogeneity of the present meta-analysis (*I*^2^ was generally between 40% and 55%), there were still some differences in study design that may have affected the robustness of the results. Through sensitivity analysis, we found that the effect size and heterogeneity did not change significantly after excluding individual studies, suggesting that the results of the current analysis are highly robust. This further enhances our confidence in the results.

By funnel plot and Egger test, no significant publication bias was found in this analysis, indicating that the literature screening process and analysis results were more reliable. This is also consistent with the results of the previous meta-analysis that support the multiple health benefits of vitamin D supplementation for patients with metabolic syndrome. Despite the high quality of the design and execution of this meta-analysis, some limitations remain. First, there were large differences in subject characteristics and interventions across studies, which may have had an impact on the heterogeneity of the analyzed results. Second, some of the studies had a short follow-up period, which did not allow for an adequate assessment of the effects of long-term vitamin D supplementation. In addition, differences in baseline vitamin D levels may have affected the interpretation of the analyzed results. Future studies should focus more on long-term, large-sample randomized controlled trials to further validate the health benefits of vitamin D supplementation.

## 5. Conclusion

This meta-analysis demonstrated that vitamin D supplementation, especially vitamin D_3_ and active vitamin D, had a was associated with a statistically significant reduction in body fat distribution and glycolipid metabolism in patients with obesity-associated metabolic syndrome. Vitamin D_3_ was outstandingly effective in reducing visceral fat, lowering fasting glucose and improving insulin resistance, and its efficacy was further enhanced by high doses and long-term interventions. In contrast, the effect of vitamin D_2_ was more limited, especially in the improvement of glucose-lipid metabolism. Patients with baseline vitamin D deficiency benefited most significantly. Despite some study heterogeneity, sensitivity analyses and publication bias detection demonstrated the robustness and reliability of the results of this analysis. Future studies should continue to focus on long-term, large-sample randomized controlled trials to further validate the health benefits of vitamin D and to clarify its optimal dosage and duration of intervention.

## Author contributions

**Conceptualization:** Qin Huang, Jidong Zhan, Yuan Gui, Ming Ma, E. Li.

**Data curation:** Qin Huang, Jidong Zhan, Yuan Gui, E. Li.

**Formal analysis:** Qin Huang, Jidong Zhan, Yuan Gui, E. Li.

**Funding acquisition:** Qin Huang, E. Li.

**Writing – original draft:** Qin Huang, Ming Ma, E. Li.

**Writing – review & editing:** Qin Huang, Ming Ma, E. Li.
